# A Comparison of the Effects of Oral Glutamine Dipeptide, Glutamine, and Alanine on Blood Amino Acid Availability in Rats Submitted to Insulin-Induced Hypoglycemia

**DOI:** 10.3390/nu6104520

**Published:** 2014-10-21

**Authors:** Vania C. Minguetti-Câmara, Any de C. R. Marques, Fabiana P. M. Schiavon, Vanessa R. Vilela, Marcos L. Brusch, Roberto Barbosa Bazotte

**Affiliations:** 1 Department of Pharmacology and Therapeutic, State University of Maringá, Maringá, Paraná State 87020-900, Brazil; E-Mails: vania@materbaby.com.br (V.C.M.-C.); anycrm@hotmail.com (A.C.R.M.); biamonteiroschiavon@gmail.com (F.P.M.S.); vilelarvanessa@hotmail.com (V.R.V.); 2 Department of Pharmacy, State University of Maringá, Maringá, Paraná State 87020-900, Brazil; E-Mail: mlbruschi@gmail.com

**Keywords:** glutaminedipeptide, aminoacidemia, glutamine, alanine, hypoglycemia, liver

## Abstract

We compared the effects of oral administration of high-dose or low-dose glutamine dipeptide (GDP), alanine (ALA), glutamine (GLN), and ALA + GLN on the blood availability of amino acids in rats submitted to insulin-induced hypoglycemia (IIH). Insulin detemir (1 U/kg) was intraperitoneally injected to produce IIH; this was followed by oral administration of GDP, GLN + ALA, GLN, or ALA. We observed higher blood levels of GLN, 30 min after oral administration of high-dose GDP (1000 mg/kg) than after administration of ALA (381 mg/kg) + GLN (619 mg/kg), GLN (619 mg/kg), or ALA (381 mg/kg). However, we did not observe the same differences after oral administration of low-dose GDP (100 mg/kg) compared with ALA (38.1 mg/kg) + GLN (61.9 mg/kg), GLN (61.9 mg/kg), or ALA (38.1 mg/kg). We also observed less liver catabolism of GDP compared to ALA and GLN. In conclusion, high-dose GDP promoted higher blood levels of GLN than oral ALA + GLN, GLN, or ALA. Moreover, the lower levels of liver catabolism of GDP, compared to ALA or GLN, contributed to the superior performance of high-dose GDP in terms of blood availability of GLN.

## 1. Introduction

The amino acid glutamine (GLN) is involved in many processes that are vital to cell function [1]. The molecular mechanisms of GLN’s actions are not entirely clear, but they undoubtedly involve changes in gene and protein expression, protein activity, and intracellular metabolite concentrations [1,2]. Enteral and parenteral administration of GLN offers nutritional benefits for critically ill patients and is recommended for use in this population [3,4,5]. Additionally, oral GLN has been used by healthy individuals, particularly athletes, to maintain immune function [6,7,8].

Although oral GLN supplementation offers potential benefits, its low solubility and stability in aqueous solutions limits its blood availability [9]. Furthermore, approximately 50% of orally administered GLN is extracted by the splanchnic bed in healthy humans [10]. However, this limitation can be overcome with the use of synthetic, stable, highly soluble glutamine dipeptide (GDP), a dipeptide composed of alanine (ALA) and GLN [11,12].

However, the performance of oral GDP in terms of blood concentration compared to oral GLN is controversial. Few studies have investigated the pharmacokinetic responses to oral doses of GDP [13,14,15].

A proper evaluation of the efficacy of GDP compared to GLN requires controlled conditions [16]. Fasted rats submitted to insulin-induced hypoglycemia (IIH) represent a suitable experimental model to evaluate changes in blood levels of amino acids [17,18]. Therefore, we compared the effects of oral GDP, GLN plus ALA, GLN, or ALA on the blood availability of amino acids in hypoglycemic rats.

## 2. Experimental Section

### 2.1. Chemicals

Insulin detemir (Levemir^®^) was obtained from Novo Nordisk (São Paulo, SP, Brazil). GDP was purchased from Ajinomoto (Ajinomoto North America, NC, USA). GLN and ALA were obtained from ICN Pharmaceuticals, Inc. (Costa Mesa, CA, USA). All other reagents were of the highest purity obtainable.

### 2.2. Animals

A total of 214 male Wistar rats, weighing approximately 250 g, were housed under a controlled 12 h light/dark cycle and a temperature of 23°C ± 2°C. All rats were fasted for 15 h before initiating the experiments. The experimental protocol was approved by the Ethics Committee of State University of Maringá, Brazil and was in accordance with international law on the protection and use of animals.

### 2.3. Experimental Protocol

We produced IIH in the rats by the intraperitoneal (ip) injection of insulin detemir (1 U/kg). We chose the dose of insulin on the basis of a previous study [18]. The insulin was not diluted, and it was injected with an infusion pump. Next, the rats received an oral administration (gavage) of GDP, ALA + GLN, GLN, or ALA immediately after ip insulin injection. An additional control group which received ip saline + oral vehicle was included. The control group furnishes the values before the administration of insulin and the test substances.

All rats were anesthetized with an ip injection of sodium thiopental (45 mg/kg). We collected blood from the vena cava to measure levels of blood glucose [19] and amino acids [20].

### 2.4. Measurement of Blood Glucose and Amino Acid Levels after Oral Administration of Low-Dose GDP (100 mg/kg), GLN (61.9 mg/kg) + ALA (38.1 mg/kg), GLN (61.9 mg/kg), or ALA (38.1 mg/kg)

An oral vehicle (IIH + oral vehicle), GDP (IIH + oral GDP), GLN + ALA (IIH + oral GLN + oral ALA), GLN (IIH + oral GLN) or ALA (IIH + oral ALA) was administered to the rats by gavage immediately after ip injection of insulin. Blood was collected 15 min (Table 1), 30 min (Table 2), and 120 min (Table 3) after oral administration to measure glucose and amino acid levels. The dose of GDP was based on a previous study [18], and the doses of ALA and GLN reflected their proportions in the GDP molecule. An additional control group received ip saline + oral vehicle.

**Table 1:** Blood glucose (mg/dL) and amino acid (nmol/mL) levels 15 min after intraperitoneal insulin injection (IIH group). The IIH group was divided into 5 subgroups: IIH + oral vehicle (VHC), IIH + oral glutamine dipeptide (GDP), IIH + oral glutamine (GLN) + oral alanine (ALA), IIH + oral GLN or IIH + oral ALA. GDP (100 mg/kg**)**, GLN (61.9 mg/kg) + ALA (38.1 mg/kg), GLN (61.9 mg/kg) or ALA (38.1 mg/kg) or VHC was administered immediately after insulin injection. The Control group received intraperitoneal saline + oral VHC. Data expressed as means ± standard error were analyzed by ANOVA (Newman-Keuls *post hoc* test). **^a^** *p*< 0.05 *vs.* Control and **^b^** *p*< 0.05 *vs.* IIH + oral GDP.

	Control (*n* = 4)	IIH + VHC (*n* = 5)	IIH + GDP (*n* = 5)	IIH+ GLN + ALA (*n* = 4)	IIH + GLN (*n* = 5)	IIH + ALA (*n* = 4)
Glucose	108.9 ± 9.0	86.2 ± 8.2	93 ± 3.0	94.2 ± 8.5	89.3 ± 3.2	90.2 ± 3.4
Alanine	704.3 ± 45.0	545.3 ± 66.7	781.1 ± 140.7	551 ± 81.2	556.6 ± 19.7	572.9 ± 89.3
Arginine	68.2 ± 19.0	26.5 ± 0.4 **^ab^**	79.5 ± 5.7	51.8 ± 14.5	57 ± 14.2	32.5 ± 2.1 **^b^**
Glutamine	844.4 ± 104.0	694.9 ± 101.2	660.8 ± 134.6	575.3 ± 121.6	775.3 ± 69.3	565.1 ± 58.2
Histidine	91 ± 1.3	90.9 ± 4.3	91.9 ± 4.0	90.3 ± 6.0	90.5 ± 6.6	82.3 ± 5.4
Isoleucine	114.5 ± 10.2	81.3 ± 10.8	114.5 ± 10.2	76.3 ± 11.0	108.6 ± 6.1	80 ± 5.9
Leucine	222 ± 16.3	165.4 ± 21.2	210.7 ± 26.6	172 ± 30.2	193.9 ± 12.2	164.1 ± 13.9
Metionine	62.6 ± 10.8	53.4 ± 6.6	63.8 ± 5.3	49.2 ± 11.2	53.7 ± 9.2	46.7 ± 3.2
Phenylalanine	94 ± 9.4	80.5 ± 9.2	91.6 ± 12.5	73 ± 11.7	87 ± 5.2	70.6 ± 3.2
Tryptophan	107.7 ± 6.8	117.9 ± 9.1	123.5 ± 15.1	94.9 ± 17.8	111.9 ± 12.9	89.1 ± 6.9
Tyrosine	111.7 ± 22.0	88.2 ± 5.6	95.9 ± 9.6	91 ± 6.8	97.6 ± 7.7	86.4 ± 10.8
Valine	255.6 ± 41.0	207.5 ± 29.7	270.5 ± 27.5	203.4 ± 31.8	263.7 ± 17.7	186.1 ± 15.7

**Table 2:** Blood glucose (mg/dL) and amino acid (nmol/mL) levels 30 min after intraperitoneal insulin injection (IIH group). The IIH group was divided into 5 subgroups: IIH + oral vehicle (VHC), IIH + oral glutamine dipeptide (GDP), IIH + oral glutamine (GLN) + oral alanine (ALA), IIH + oral GLN or IIH + oral ALA. GDP (100 mg/kg), GLN (61.9 mg/kg) + ALA (38.1 mg/kg), GLN (61.9 mg/kg) or ALA (38.1 mg/kg) or VHC. The Control group received intraperitoneal saline + oral VHC. Data expressed as means ± standard error were analyzed by ANOVA (Newman-Keuls *post hoc* test).

	Control (*n* = 4)	IIH + VHC (*n* = 5)	IIH + GDP (*n* = 5)	IIH+ GLN + ALA (*n* = 4)	IIH + GLN (*n* = 5)	IIH + ALA (*n* = 4)
Glucose	105.9 ± 15.1	71.6 ± 21.3	82.5 ± 21.5	74.0 ± 14.9	68.6 ± 14.9	80.8 ± 10.6
Alanine	272.5 ± 94.4	243.9 ± 55.1	260.8 ± 60.7	304.9 ± 77.9	250.7 ± 31.0	287.5± 86.0
Arginine	66.8 ± 22.6	51.3 ± 19.6	49.2 ± 19.5	54.9 ± 15.1	46.6 ± 12.5	46.7 ± 12.4
Glutamine	367.9 ± 77.3	305.8 ± 57.5	282.3 ± 57.1	339.0 ± 68.8	327.7 ± 76.0	325.2 ± 85.0
Histidine	85.3 ± 7.9	90.2 ± 7.8	88.9 ± 9.5	88.3 ± 5.5	90.6 ± 8.2	91.4 ± 7.2
Isoleucine	83.7 ± 16.5	59.9 ± 17.0	54.6 ± 13.5	52.2 ± 14.2	48.3 ± 13.7	51.0 ± 9.0
Leucine	157.8 ± 33.0	115.5 ± 37.2	103.7 ± 19.9	104.7 ± 22.6	95.1 ± 19.9	101.0 ± 18.5
Metionine	37.3 ± 12.9	35.1 ± 3.4	33.9 ± 5.5	36.8 ± 6.5	37.0 ± 4.2	35.9 ± 11.9
Phenylalanine	69.2 ± 15.2	60.0 ± 12.8	54.3 ± 7.1	59.7 ± 11.0	56.1 ± 8.4	55.1 ± 11.4
Tryptophan	60.3 ± 19.9	76.2 ± 12.3	58.9 ± 11.2	60.9 ± 12.0	74.7 ± 13.0	60.8 ± 19.7
Tyrosine	77.8 ± 13.3	73.3 ± 16.7	74.5 ± 18.6	75.5 ± 18.7	73.0 ± 22.5	83.0 ± 16.5
Valine	180.8 ± 32.8	145.9 ± 34.7	134.7 ± 24.9	140.7 ± 23.5	127.4 ± 19.2	131.6 ± 15.7

**Table 3:** Blood glucose (mg/dL) and amino acid (nmol/mL) levels 120 min after intraperitoneal insulin injection (IIH group). The IIH group was divided into 5 subgroups: IIH + oral vehicle (VHC), IIH + oral glutamine dipeptide (GDP), IIH + oral glutamine (GLN) + oral alanine (ALA), IIH + oral GLN or IIH + oral ALA. GDP (100 mg/kg), GLN (61.9 mg/kg) + ALA (38.1 mg/kg), GLN (61.9 mg/kg), or ALA (38.1 mg/kg) or VHC was orally administered. The Control group received intraperitoneal saline + oral VHC. Data expressed as means ± standard error were analyzed by ANOVA (Newman-Keuls *post hoc* test). **^a ^***p*< 0.05 *vs.* Control and **^b^** *p*< 0.05 *vs*. IIH + oral GDP.

	Control (*n* = 4)	IIH + VHC (*n* = 5)	IIH +GDP (*n* = 5)	IIH+ GLN + ALA (*n* = 4)	IIH + GLN (*n* = 5)	IIH + ALA (*n* = 4)
Glucose	87.9 ± 4.3	18.0 ± 1.7 **^a^**	20.8 ± 7.4 **^a^**	33.3 ± 17.0 **^a^**	23.2 ± 5.9 **^a^**	15.0 ± 1.4 **^a^**
Alanine	607.8 ± 39.8	457.6± 31.3	353.0 ± 33.3	470.4 ± 97.6	464.8 ± 36.7	506.9± 45.5
Arginine	111.8 ± 26.4	103.1± 12.2	101.7 ± 1.9	97.9 ± 18.2	102.9 ± 17.0	94.4 ± 16.5
Glutamine	871.7 ± 93.2	613.3± 38.0	463.7± 68.1 **^a^**	647.5 ±127.9	516.0 ±87.2 **^a^**	459.2 ±65.8 **^a^**
Histidine	88.6 ± 3.3	93.5 ± 4.9	85.3 ± 6.6	85.2 ± 3.7	88.8 ± 2.5	93.5 ± 1.6
Isoleucine	109.1 ± 6.3	58.2 ± 5.6 **^a^**	59.5 ± 6.1 **^a^**	73.7 ± 19.4 **^a^**	53.34 ± 4.4 **^a^**	62.5 ± 2.3 **^a^**
Leucine	196.3 ± 10.9	124.8± 9.6 **^a^**	118.5± 10.3 **^a^**	130.4 ±27.8 **^a^**	109.5 ± 8.9 **^a^**	136.4± 12.5 **^a^**
Metionine	54.1 ± 6.0	38.4 ± 4.0	23.2 ± 3.9 **^a^**	43.9 ± 9.4 **^b^**	45.1 ± 3.7 **^c^**	33.0 ± 3.6
Phenylalanine	85.0 ± 3.4	82.9 ± 4.4	69.8 ± 5.5	80.9 ± 5.6	75.8 ± 3.7	83.7 ± 5.7
Tryptophan	99.0 ± 6.4	175.1±20.5 **^a^**	121.5 ± 11.4	139.2 ± 19.4	156.2 ± 14.0	145.2 ± 11.6
Tyrosine	110.2 ± 10.7	99.7 ± 8.7	99.9 ± 2.4	96.6 ± 15.0	95.7 ± 12.4	109.0 ± 4.2
Valine	295.7 ± 18.9	164.5±10.1 **^a^**	152.4 ± 16.4 **^a^**	196.9 ±34.2 **^a^**	160.3 ±13.4 **^a^**	185.4 ± 6.3 **^a^**

### 2.5. Measurement of Blood Glucose and Amino Acid Levels after Oral Administration of High-Dose GDP (1000 mg/kg), GLN (619 mg/kg) + ALA (381 mg/kg), GLN (619 mg/kg), or ALA (381 mg/kg)

An oral vehicle (IIH + oral vehicle), GDP (IIH + oral GDP), GLN + ALA (IIH + oral GLN + oral ALA), GLN (IIH + oral GLN) or ALA (IIH + oral ALA) was administered to the rats by gavage immediately after ip insulin injection. Blood was collected 15 min (Table 4) and 30 min (Table 5) after oral administration to measure glucose and amino acid levels. An additional control group received ip saline + oral vehicle. The doses of ALA and GLN reflected their proportions in the GDP molecule.

### 2.6. Liver Perfusion Experiments

The hepatic catabolism of GDP, GLN, and ALA, inferred from liver glucose and urea formation were evaluated. Liver perfusion experiments were conducted in an open system without recirculation of perfusate, as previously described [17]. The rats were anesthetized with an ip injection of sodium thiopental (45 mg/g). The abdomen was opened by midline incision and a cannula was inserted into the portal vein.

The perfusion fluid, Krebs-Henseleit buffer (KHB; pH 7.4), which was saturated with an oxygen/carbon dioxide (95%/5%) mixture, was pumped (4 ml/g·min of liver weight) through a temperature-regulated (37 °C) membrane oxygenator prior to entering the liver via the cannula inserted in the portal vein.

**Table 4:** Blood glucose (mg/dL) and amino acid (nmol/mL) levels 15 min after intraperitoneal insulin injection (IIH group). The IIH group was divided into 5 subgroups: IIH + oral vehicle (VHC), IIH + oral glutamine dipeptide (GDP), IIH + oral glutamine (GLN) + oral alanine (ALA), IIH + oral GLN or IIH + oral ALA. GDP (1000 mg/kg), GLN (619 mg/kg) + ALA (381 mg/kg), GLN (619 mg/kg), ALA (381 mg/kg), or VHC was administered. The Control group received intraperitoneal saline + oral VHC. Data expressed as means ± standard error were analyzed by ANOVA (Newman-Keuls *post hoc* test).

	Control (*n* = 5)	IIH + VHC (*n* = 5)	IIH + GDP (*n* = 5)	IIH+ GLN + ALA (*n* = 3)	IIH + GLN (*n* = 5)	IIH + ALA (*n* = 5)
Glucose	134.2 ± 9.2	128.1 ± 6.2	145.8 ± 11.7	126.7 ± 9.9	126.8 ± 5.3	118.1 ± 6.4
Alanine	532.1 ± 23.8	606 ± 104.6	1477 ± 601.2	792.3 ± 150	688.9 ± 65.3	1001.6 ± 172
Arginine	104.2 ± 13.1	146.9 ± 17.6	157.1 ± 32.5	94.3 ± 8.5	120.9 ± 24.2	118.4 ± 12.7
Glutamine	622 ± 33.3	608.2 ± 40.7	1052.7 ± 334	514.2 ± 101	853.7 ± 173	650 ± 14.0
Histidine	91.3 ± 4.5	94.5 ± 10.1	108.4 ± 17.6	87 ± 3.4	91.1 ± 8.0	85.1 ± 2.2
Isoleucine	78.8 ± 4.9	74 ± 5.9	69 ± 8.3	50.1 ± 0.9	65.6 ± 7.2	78.4 ± 3.3
Leucine	149.5 ± 13.2	138 ± 17.9	138 ± 20.6	102 ± 7.8	128.2 ± 14.8	141.8 ± 12.3
Metionine	51.2 ± 4.6	59 ± 7.1	49.2 ± 4.1	38.4 ± 6.5	47.2 ± 5.0	57.3 ± 6.7
Phenylalanine	61.3 ± 3.8	63.7 ± 8.6	67.8 ± 9.0	46.4 ± 3.8	58.7 ± 6.1	58.6 ± 3.5
Tryptophan	73.7 ± 6.0	100 ± 5.9	90.7 ± 3.8	68.9 ± 11.1	88.8 ± 12.1	85.3 ± 5.5
Tyrosine	135.3 ± 14.0	153.4 ± 17.2	210.1 ± 49.3	140.1 ± 28.8	143.8 ± 18.6	177.9 ± 27.5
Valine	193.9 ± 3.9	185.6 ± 8.5	172.2 ± 17.9	136.5 ± 6.3	171.9 ± 22.6	197.5 ± 10.3

**Table 5:** Blood glucose (mg/dL) and amino acid (nmol/mL) levels 30 min after intraperitoneal insulin injection (IIH group). The IIH group was divided into 5 subgroups: IIH + oral vehicle (VHC), IIH + oral glutamine dipeptide (GDP), IIH + oral glutamine (GLN) + oral alanine (ALA), IIH + oral GLN or IIH + oral ALA. GDP (1000 mg/kg**)**, GLN (619 mg/kg) + ALA (381 mg/kg), GLN (619 mg/kg) or ALA (381 mg/kg) or VHC was administered immediately after insulin injection. The Control group received intraperitoneal saline + oral VHC. Data expressed as means ± standard error were analyzed by ANOVA (Newman-Keuls *post hoc* test). **^a^** *p* < 0.05 *vs.* Control, **^b^** *p* < 0.05 *vs.* IIH + VHC and **^c^** *p* < 0.05 *vs.* IIH + oral GDP.

	Control (*n* = 5)	IIH + VHC (*n* = 5)	IIH + GDP (*n* = 5)	IIH+ GLN + ALA (*n* = 4)	IIH +GLN (*n* = 5)	IIH + ALA (*n* = 5)
Glucose	102.4 ± 6.3	43.1 ± 6.8 **^a^**	80.1 ± 3.7 **^ab^**	67.4 ± 6.7 **^ab^**	69.2 ±4.2 **^ab^**	55.0 ± 6.8 **^a^**
Alanine	373.3 ± 19.4	358.3 ± 16.7	1318±73.3 **^ab^**	926.3± 90.5 **^ab^**	486.1 ± 0.8	959 ± 116 **^ab^**
Arginine	84.1 ± 14.0	67.1 ± 10.61	99.4 ± 19.1	76.9 ± 415.2	118.5 ± 9.2	76.0 ± 6.5
Glutamine	686.7 ± 10.0	589 ± 31.8	1225±63.1 **^ab^**	842.7± 87.8**^c^**	1006±87.2 **^abc^**	666.8 ± 48.7 **^c^**
Histidine	86.1 ± 5.8	99 ± 3.3	101.6 ± 8.6	92.4 ± 5.8	91.3 ± 5.3	93.4 ± 2.1
Isoleucine	107.1± 17.8	45.9 ± 5.9 **^a^**	55.4 ± 2.5 **^a^**	61.4 ± 6.7 **^a^**	58.4 ± 7.2 **^a^**	44.9 ± 2.4 **^a^**
Leucine	134 ± 5.4	83 ± 8.5 **^a^**	89.9 ± 6.4 **^a^**	97.8 ± 10.7 **^a^**	89.4 ± 10.7 **^a^**	75.6 ± 3.4 **^a^**
Metionine	40.2 ± 4.6	41.1 ± 4.2	36.1 ± 2.2	45.1 ± 3.3	37.4 ± 4.0	38.9 ± 2.9
Phenylalanine	64.5 ± 2.0	55.4 ± 3.1	51.3 ± 2.7 **^a^**	59.6 ± 2.8	51.6 ± 5.1 **^a^**	49.6 ± 0.5 **^a^**
Tryptophan	69.4 ± 1.8	81.6 ± 5.2	68.7 ± 7.0	82.4 ± 5.0	68.2 ± 7.3	80.1 ± 2.3
Tyrosine	97.7 ± 6.5	85.4 ± 5.1	129.9 ± 16.3	115.1 ± 7.1	104.8 ± 21.8	92.6 ± 6.7
Valine	162.6 ± 7.1	113.2 ± 10.2 **^a^**	121 ± 7.1 **^a^**	137.8 ± 11.9	116.2± 12.1 **^a^**	114 ± 5.0 **^a^**

**Figure 1:**
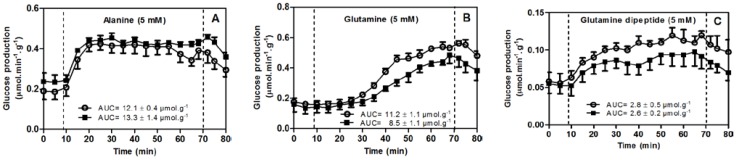
Glucose production from alanine (**A**); glutamine (**B**); and glutamine dipeptide (**C**) in livers from rats fasted for 15 h. Livers were perfused as described in the experimental section. Experiments were performed 30 min after intraperitoneal administration of saline (Control group, ■) or insulin detemir (Hypoglycemic group, **○**). Data are expressed as means ± standard error of 4 experiments. AUC = area under the curves.

**Figure 2:**
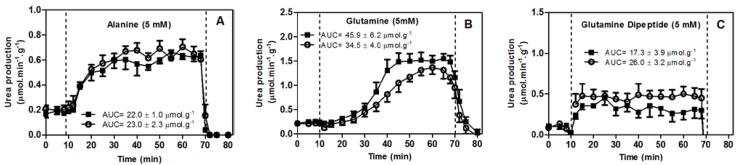
Urea production from alanine (**A**); glutamine (**B**); and glutamine dipeptide (**C**) in livers from rats fasted for 15 h. Livers were perfused as described in the experimental section. Experiments were performed 30 min after intraperitoneal administration of saline (Control group, ■) or insulin detemir (Hypoglycemic group, **○**). Data are expressed as means ± standard error of 4 experiments. AUC = area under the curve.

The concentrations of ALA (5 mM), GLN (5 mM), or GDP (5 mM) to be dissolved in the perfusion fluid were chosen on the basis of a previous study [18]. As shown in Figure 1 and Figure 2, after a pre-infusion period (10 min), ALA, GLN, or GDP was dissolved in KHB and infused over 60 min. This was followed by a post-infusion period (10 min) to allow the return to basal values of the pre infusion period. Samples of the effluent perfusion fluid were obtained at 5 min intervals and the glucose [19] and urea [21] concentrations were measured. Glucose and urea formation indicated liver catabolism of ALA, GLN, and GDP. The areas under the curves (AUC) of the infusion periods are expressed as μmol/g.

### 2.7. Statistical Procedures

Data were analyzed by ANOVA (Newman-Keuls *post hoc* test) using Graph-Pad Prism Version 5.0 software (GraphPad Software, Inc., La Jolla, USA). Results are reported as means ± standard error of the means. *p*-values < 0.05 indicated statistical significance.

## 3. Results

Oral administration of low-dose GDP (100 mg/kg), GLN (61.9 mg/kg) + ALA (38.1 mg/kg), GLN (61.9 mg/kg), or ALA (38.1 mg/kg) did not change blood concentrations of GLN, ALA, histidine, valine, leucine, isoleucine, methionine, phenylalanine, tryptophan and tyrosine at 15 min (Table 1), 30 min (Table 2), or 120 min (Table 3) after insulin injection.

In contrast with the low-dose GDP, blood concentrations of GLN tended to be higher 15 min after administration of high-dose GDP (1000 mg/kg) compared to GLN (619 mg/kg) + ALA (381 mg/kg), GLN (619 mg/kg) or ALA (381 mg/kg) (Table 4). However, we observed substantial variability in GLN blood levels, so we repeated the experiments to measure blood amino acid levels 30 min after oral administration of high-dose GDP, GLN + ALA, GLN, or ALA.

After 30 min, IIH rats that received oral GDP (1000 mg mg/kg) showed higher (*p* < 0.05) blood levels of GLN compared to GLN (619 mg/kg) + ALA (381 mg/kg), GLN (619 mg/kg) or ALA (381 mg/kg) (Table 5).

On the other hand, the blood levels of arginine, histidine, valine, leucine, isoleucine, methionine, phenylalanine, tryptophan and tyrosine remained unchanged after oral administration of low-dose (Table 4) or high-dose (Table 5) of GDP, GLN + ALA, GLN, or ALA.

Since increased levels of GLN occurred 30 min after the administration of high-dose GDP, GLN + ALA, GLN or ALA, this time period was chosen to evaluate how much the liver catabolism of GDP, GLN or ALA, which were inferred from liver glucose and urea formation, contributed to blood levels of GLN or ALA. From these experiments, we observed more intense liver catabolism during the infusion of ALA (Figure 1A and Figure 2A for glucose and urea, respectively) and GLN (Figure 1B and Figure 2B for glucose and urea, respectively) compared to the infusion of GDP (Figure 1C and Figure 2C for glucose and urea, respectively).

## 4. Discussion

Hypoaminoacidemia is a common feature of IIH [22,23,24,25] and involves the inhibition of proteolysis and the stimulation of amino acid uptake in liver and muscle [26]. We observed decreased (*p* < 0.05) blood concentrations of branched chain amino acids (BCAA), such as valine, leucine and isoleucine, 30 min (Table 5) after insulin injection, which agrees with results of previous study [17]. In addition, high-dose oral GDP (1000 mg/kg), GLN (619 mg/kg) + ALA (381 mg/kg), GLN (619 mg/kg), and ALA (381 mg/ kg) did not overcome the reduced blood levels of BCAA produced by insulin injection (Table 5).

Oral GDP (1000 mg/kg) performed better in terms of blood GLN availability than oral GLN (619 mg/kg) (Table 5). This could be because the enterocytes utilize GLN at high rates [1,2], which reduces its availability for intestinal absorption. This is reinforced by the fact that parenteral administration of GLN, which overcomes the influence of intestinal metabolism, showed better glycemia recovery than oral GLN in IIH rats [17].

It should be emphasized that the integrity of the GDP molecule is needed to obtain this effect. Blood GLN availability was lower (*p* < 0.05) with combined oral ALA (381 mg/kg) + GLN (619 mg/kg) than with oral GDP (1000 mg/kg) (Table 5).

The primary intestinal mechanism for GDP assimilation is its absorption as an intact dipeptide rather than by hydrolysis [15]. Additionally, it is difficult to estimate exactly how much oral GDP, GLN, or ALA enters the liver. Therefore, we compared the hepatic catabolism of these substances. Since GLN and ALA showed higher blood levels 30 min (Table 5) after the administration of GDP (1000 mg/kg), GLN (619 mg/kg) + ALA (381 mg/kg), GLN (619 mg/kg), or ALA (381 mg/kg), we chose this time period to evaluate how much the liver catabolism of GDP, GLN, or ALA, which we inferred from liver glucose and urea production, contributed to blood levels of GLN and ALA.

It is noteworthy that the increase in liver catabolism was smaller with GDP (Figure 1C and Figure 2C) than with ALA (Figure 1A and Figure 2A) or GLN (Figure 2B and Figure 2B) in rats that received the vehicle (control group) and in rats that received insulin (IIH group). The reason for this difference is the fact that the hepatocytes do not have a transport system for dipeptides; hepatocytes assimilate dipeptides by extracellular hydrolysis from enzymes located on the plasma membranes following its release into the cytosol as ALA and GLN [27]. Therefore, like enterocytes, hepatocytes showed less intense catabolism with GDP than with GLN.

Less intense catabolism of GDP compared to ALA and GLN occurs not only in the enterocytes [28] but also in the liver (Figure 1 and Figure 2). Additionally, blood GDP is rapidly hydrolyzed [29] and liberated GLN is used for energy production [30]. These facts help explain why high-dose oral GDP yields superior blood availability of amino acids than oral GLN + ALA, GLN, or ALA.

Taken together, these results have great clinical interest because they help one to understand the performance of oral GDP and oral GLN in terms of blood concentration of GLN.

## 5. Conclusions

In conclusion, our results indicate that the oral administration of high-dose GDP (1000 mg/kg) displayed better performance than oral GLN (619 mg/kg) + ALA (381 mg/kg), GLN (619 mg/kg), or ALA (381 mg/kg) in terms of blood availability of GLN. No differences were observed in blood availability of ALA or GLN after the administration of low-dose GDP (100 mg/kg).
